# Endotoxin Hemoperfusion as an Adjuvant Therapy for Sepsis: Lessons from the TIGRIS Trial

**DOI:** 10.14789/ejmj.JMJ25-0043-R

**Published:** 2025-12-10

**Authors:** YU-CHANG YEH, KUNIHIKO NAGAKARI, TOSHIAKI IBA

**Affiliations:** 1 Department of Anesthesiology, National Taiwan University Hospital, Taipei, Taiwan; 1 Department of Anesthesiology, National Taiwan University Hospital, Taipei, Taiwan; 2 Department of Surgery, Juntendo University Urayasu Hospital, Chiba, Japan; 2 Department of Surgery, Juntendo University Urayasu Hospital, Chiba, Japan; 3 Faculty of Medical Science, Juntendo University, Chiba, Japan; 3 Faculty of Medical Science, Juntendo University, Chiba, Japan

**Keywords:** sepsis, polymyxin B, shock, organ dysfunction, randomized controlled trial

## Abstract

Despite significant advances in antimicrobial therapy and supportive intensive care, mortality in septic shock remains unacceptably high. Hemoadsorption therapies have emerged as adjunctive strategies designed to remove circulating mediators that propagate the dysregulated host response. Among these, polymyxin B hemoperfusion (PMX-HP) represents the most extensively studied extracorporeal modality, specifically targeting circulating endotoxin. Over three decades of investigation have produced a complex body of evidence, ranging from early promising results in small, open-label studies to large randomized trials with inconsistent outcomes. Recent findings from the TIGRIS trial, employing Bayesian design and biomarker-guided patient selection, provide compelling support for a survival benefit in a well-defined subgroup of septic shock patients with intermediate endotoxin activity. This review synthesizes the evolution of PMX-HP research, from EUPHAS through EUPHRATES and TIGRIS, highlighting lessons learned in trial design, biomarker utilization, and patient stratification. These experiences underscore the potential of precision-based extracorporeal interventions in sepsis while outlining the critical methodological and regulatory challenges that remain.

## Introduction

Sepsis remains one of the most challenging syndromes in modern intensive care, a disorder in which the body’s immune response to infection becomes so dysregulated that it threatens its own survival. Despite decades of effort, the central paradox of sepsis treatment persists: while antibiotics can effectively eradicate pathogens, they cannot neutralize the toxins released in the process. Among these, endotoxin (lipopolysaccharide [LPS]) stands out as a critical trigger of inflammation, endothelial dysfunction, and multiorgan failure. The recognition that conventional therapies cannot easily control the toxic aftermath of bacterial death led clinicians and engineers to explore the possibility of physically removing endotoxin from the bloodstream. Polymyxin B hemoperfusion (PMX-HP) was developed to address this limitation by selectively adsorbing circulating endotoxin^1-4)^. Over the past 30 years, the clinical evaluation of PMX-HP has progressed through several pivotal trials─EUPHAS^5)^, ABDO- MIX^6)^, EUPHRATES^7)^, and TIGRIS^8)^, each contributing essential insights into the mechanisms, limitations, and potential of endotoxin adsorption therapy.

## Mechanistic basis of polymyxin B hemoperfusion

The concept of hemoadsorption emerged from this idea, an extracorporeal technique in which blood passes through a cartridge containing adsorptive material capable of binding deleterious molecules^3)^. Among various approaches, polymyxin B hemoperfusion (PMX-HP) is the most mature and best-studied. Its origin dates back to Japan in the late 1980s, when researchers discovered that immobilizing polymyxin B, an antibiotic known for its strong affinity to the lipid A domain of endotoxin, on polystyrene fibers allowed selective removal of circulating endotoxin without the nephrotoxicity that limited its systemic use^4)^. The resulting device, later commercialized as Toraymyxin™, was introduced into clinical practice in 1994 and has since been used in hundreds of thousands of cases worldwide, most extensively in Japan and Europe^4, 9)^.

The biological rationale for PMX-HP is compelling. When endotoxin binds to Toll-like receptor 4 on monocytes and endothelial cells, it triggers an explosive release of cytokines and a cascade of microvascular injury^1, 2)^. By physically extracting endotoxin from the circulation, PMX-HP interrupts this signaling loop at its source. Experimental studies have shown that hemoperfusion through the polymyxin column can reduce plasma endotoxin activity and proinflammatory cytokines, stabilize vascular tone, improve microcirculation, and mitigate organ failure^7, 9, 10)^. Early clinical experience in septic shock, particularly in patients with intra-abdominal infections, was often dramatic, with marked improvements in blood pressure and urine output observed within hours^5, 6)^.

## Clinical evidence: Lessons from major PMX-HP trials

### Early promise: EUPHAS

The enthusiasm generated by these early reports culminated in the EUPHAS trial in 2009, which represented the first multicenter randomized evaluation of PMX-HP. Conducted in ten Italian ICUs, EUPHAS enrolled patients with abdominal septic shock following emergency surgery and compared conventional therapy with or without two sessions of PMX-HP. The results were impressive: mean arterial pressure and oxygenation improved significantly, vasopressor dependence decreased, and 28- day mortality fell from 53 % to 32 %. Although the study was terminated early at the interim analysis and thus potentially overstated the treatment effect, it established proof of principle that endotoxin adsorption could translate into tangible clinical benefit.

### Disappointment and debate: ABDO-MIX

Yet, as often happens in critical-care research, success proved difficult to reproduce. The subsequent French ABDO-MIX trial, involving more than 200 patients, failed to demonstrate a survival benefit. Several factors likely contributed: frequent cartridge clotting led to incomplete treatment in many cases, patient selection was not guided by endotoxin levels, and the lack of blinding may have introduced bias. In retrospect, it became evident that a substantial fraction of enrolled patients had little or no endotoxemia, an inclusion error that diluted any potential efficacy signal. ABDO-MIX therefore underscored a key lesson: PMX-HP does not work in the absence of its molecular target.

### Rigorous evaluation: EUPHRATES

The principle guided the design of the next major investigation, the EUPHRATES trial, launched in North America in 2010. It was the largest and most rigorous study of PMX-HP to date, using a double-blind, sham-controlled design and enrolling patients with confirmed endotoxin activity of at least 0.60 as measured by the Endotoxin Activity Assay (EAA). Despite this careful approach, the primary analysis again showed no mortality benefit. However, a *post-hoc* examination of the data revealed an intriguing pattern: patients with intermediate endotoxin activity (EAA 0.60-0.89) and substantial organ dysfunction (MODS [multiple organ dysfunction score] > 9) appeared to benefit, whereas those with extremely high endotoxin levels did not. The latter group may have represented patients in whom microvascular injury was already irreversible. This finding shifted the conceptual framework from a “one-size-fits-all” intervention to a precision-based therapy aimed at a biologically defined subgroup.

### Precision in design: TIGRIS

Out of that realization was born the TIGRIS trial, which can be viewed as both a continuation and a refinement of EUPHRATES. Adopting an adaptive Bayesian design, TIGRIS prospectively restricted enrollment to patients with EAA 0.60-0.89 and MODS > 9, precisely the cohort that had shown promise in post-hoc analysis. When the results were released in 2025, they marked a turning point. Among 151 evaluable patients, the probability that PMX-HP improved 28-day survival exceeded 95 %, with an adjusted odds ratio of 0.67 and an absolute risk reduction of 6.4 %. More strikingly, at 90 days, the benefit grew, with mortality of 43 % in the PMX group versus 61 % in controls, an absolute reduction of 17 % and a number needed to treat of just eight. For the first time, a late-phase randomized trial offered credible, statistically validated evidence that targeted endotoxin adsorption could save lives.

## Synthesis of evidence from meta-analyses

When viewed in aggregate, the sequence of EUPHAS, ABDO-MIX, EUPHRATES, and TIGRIS tells a coherent story of scientific evolution^5-8)^. Initial optimism based on small, unblinded studies gave way to skepticism after larger negative trials, only for the therapy to re-emerge—refined, more precise, and supported by robust Bayesian evidence^8, 11)^. The apparent contradictions dissolve once patient selection, disease severity, and biological phenotype are considered. PMX-HP does not benefit all patients with sepsis; rather, it benefits those in whom endotoxin drives the pathophysiology and who remain within the window of reversibility^7, 8, 12)^.

Meta-analyses over the past decade have reflected this gradual maturation. Early pooled analyses found no overall survival effect, largely due to heterogeneity across regions and inclusion criteria^13, 14)^. More recent network meta-analyses suggest a consistent trend toward benefit in high-risk, endotoxin- positive populations, although the certainty of evidence remains low^15)^. Real-world registry data have also contributed to the picture: the EUPHAS-2 and J-SEPTIC databases, encompassing hundreds of patients, confirmed that PMX-HP is feasible, safe, and associated with improved hemodynamics and reduced mortality when used appropriately^16, 17)^.

Together, these complementary strands of evidence converge on a pragmatic conclusion: the success of hemoadsorption hinges less on the device itself and more on its biological alignment with the patient’s disease stage^9, 18, 19)^.[Fig g001]

**Figure 1 g001:**
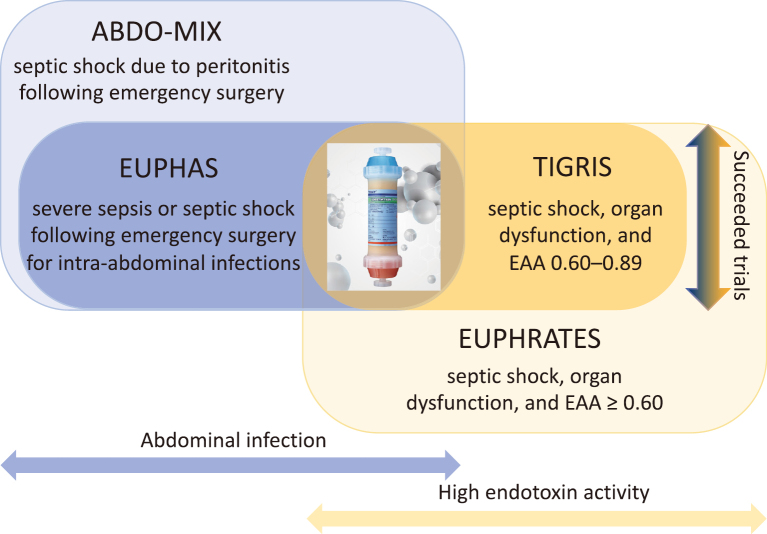
The patient population of the clinical trials The earlier clinical studies targeted septic shock due to peritonitis. The later studies limited the target population to the cases suspected to have a high endotoxin level by using the endotoxin activity assay (EAA).

## Challenges and future directions

Despite these advances, several challenges continue to limit the widespread adoption of PMX-HP. Rapid measurement of endotoxin activity is not yet universally available, constraining timely patient identification^7, 8, 11)^. The logistics of performing hemoperfusion─specialized cartridges, trained staff, and coordination with other critical-care interventions─require institutional commitment^4, 9, 16)^. Moreover, cost-effectiveness remains a key consideration in healthcare systems that demand demonstrable value for new technologies^9, 17)^. Regulatory approval outside Japan and Europe will depend on peer-reviewed publication of the TIGRIS results and confirmatory post-marketing studies^8, 11)^.

Looking ahead, the trajectory of PMX-HP and related hemoadsorption technologies points toward an era of precision extracorporeal therapy. Future research will likely integrate rapid bedside diagnostics, immune phenotyping, and dynamic monitoring of mediator levels and microcirculatory status to determine not only who should be treated but also when and for how long^10)^. Hybrid cartridges capable of adsorbing multiple classes of mediators or systems that combine endotoxin removal with cytokine modulation are already under investigation^3)^. The ultimate goal is to transform sepsis care from reactive resuscitation to mechanism-directed intervention^1, 19)^.

## Conclusion

In summary, hemoadsorption has evolved from an experimental curiosity into a credible adjunct in the management of endotoxic septic shock. The lessons of PMX-HP are instructive: early enthusiasm must be tempered by rigorous methodology, and success depends on aligning intervention with biology. The recent Bayesian evidence from TIGRIS revives confidence in the therapy’s potential. It also reminds us that sepsis is not a uniform disease; it is a syndrome of diverse molecular phenotypes. As our understanding deepens, therapies like PMX-HP may finally realize their promise as precision tools capable of restoring immune homeostasis in one of medicine’s most formidable adversaries.

## Data Availability

Not applicable.

## Author contributions

YCY and TI wrote the manuscript. KN revised the text. All authors read and approved the final manuscript.

## Conflicts of interest statement

TI, a member of the JMJ Editorial Board, was not involved in the peer review or decision-making process for this paper. The authors declare that they have no conflict of interest.

## References

[1] Kellum JA, Ronco C: The role of endotoxin in septic shock. Crit Care, 2023; 27: 400. 37858258 10.1186/s13054-023-04690-5 PMC10585761

[2] Brandenburg K, Schromm AB, Weindl G, et al: An update on endotoxin neutralization strategies in Gram-negative bacterial infections. Expert Rev Anti Infect Ther, 2021; 19: 495-517. 33210958 10.1080/14787210.2021.1834847

[3] Ronco C: Endotoxin removal: history of a mission. Blood Purif. 2014; 37 Suppl 1: 5-8. 24457488 10.1159/000356831

[4] Shimizu T, Miyake T, Tani M: History and current status of polymyxin B-immobilized fiber column for treatment of severe sepsis and septic shock. Ann Gastroenterol Surg, 2017; 1: 105-113. 29863114 10.1002/ags3.12015 PMC5881300

[5] Cruz DN, Antonelli M, Fumagalli R, et al: Early use of polymyxin B hemoperfusion in abdominal septic shock: the EUPHAS randomized controlled trial. JAMA, 2009; 301: 2445-2452. 19531784 10.1001/jama.2009.856

[6] Payen DM, Guilhot J, Launey Y, et al: Early use of polymyxin B hemoperfusion in patients with septic shock due to peritonitis: a multicenter randomized control trial. Intensive Care Med, 2015; 41: 975-984. 25862039 10.1007/s00134-015-3751-z PMC4477725

[7] Dellinger RP, Bagshaw SM, Antonelli M, et al: Effect of Targeted Polymyxin B Hemoperfusion on 28-Day Mortality in Patients With Septic Shock and Elevated Endotoxin Level: The EUPHRATES Randomized Clinical Trial. JAMA, 2018; 320: 1455-1463. 30304428 10.1001/jama.2018.14618 PMC6233793

[8] Iba T, Klein DJ: The wind changed direction and the big river still flows: from EUPHRATES to TIGRIS. J Intensive Care, 2019; 7: 31. 31131109 10.1186/s40560-019-0386-0 PMC6521478

[9] Shoji H, Ferrer R: Potential survival benefit and early recovery from organ dysfunction with polymyxin B hemoperfusion: perspectives from a real-world big data analysis and the supporting mechanisms of action. J Anesth Analg Crit Care, 2022; 2: 27. 37386673 10.1186/s44158-022-00056-5 PMC9207853

[10] Chen SH, Chan WS, Liu CM, et al: Effects of endotoxin adsorber hemoperfusion on sublingual microcirculation in patients with septic shock: a randomized controlled trial. Ann Intensive Care, 2020; 10: 80. 32533380 10.1186/s13613-020-00699-z PMC7290141

[11] Spectral Medical Inc. Spectral Medical and Vantive announce topline results from Spectral’s TIGRIS trial evaluating PMX hemoadsorption therapy for endotoxic septic shock. https://spectraldx.com/spectral-medical-and-vantive-announce-topline-results-from-spectrals-tigris-trial-evaluating-pmx-hemoadsorption-therapy-for-endotoxic-septic-shock/ (Accessed Oct 2025).

[12] Klein DJ, Foster D, Walker PM, Bagshaw SM, Mekonnen H, Antonelli M: Polymyxin B hemoperfusion in endotoxemic septic shock patients without extreme endotoxemia: a post hoc analysis of the EUPHRATES trial. Intensive Care Med, 2018; 44: 2205-2212. 30470853 10.1007/s00134-018-5463-7 PMC6280819

[13] Fujii T, Ganeko R, Kataoka Y, et al: Polymyxin B-immobilized hemoperfusion and mortality in critically ill adult patients with sepsis/septic shock: a systematic review with meta-analysis and trial sequential analysis. Intensive Care Med, 2018; 44: 167-178. 29204670 10.1007/s00134-017-5004-9

[14] Putzu A, Schorer R, Lopez-Delgado JC, Cassina T, Landoni G: Blood purification and mortality in sepsis and septic shock: a systematic review and meta-analysis of randomized trials. Anesthesiology, 2019; 131: 580-593. 31246600 10.1097/ALN.0000000000002820

[15] Chen JJ, Lai PC, Lee TH, Huang YT: Blood purification for adult patients with severe infection or sepsis/septic shock: a network meta-analysis of randomized controlled trials. Crit Care Med, 2023; 51: 1777-1789. 37470680 10.1097/CCM.0000000000005991 PMC10645104

[16] Cutuli SL, Artigas A, Fumagalli R, et al: Polymyxin-B hemoperfusion in septic patients: analysis of a multicenter registry. Ann Intensive Care, 2016; 6: 77. 27502196 10.1186/s13613-016-0178-9 PMC4977232

[17] Nakamura Y, Kitamura T, Kiyomi F, et al: Potential survival benefit of polymyxin B hemoperfusion in patients with septic shock: a propensity-matched cohort study. Crit Care, 2017; 21: 134. 28592318 10.1186/s13054-017-1712-3 PMC5463489

[18] Iba T, Helms J, Nagaoka I, Mineshima M, Ferrer R: TIGRIS and EUPHRATES eventually join and provide new evidence: a narrative review of the polymyxin B hemoperfusion. J Intensive Care. In press. 10.1186/s40560-025-00835-6 PMC12751791 41291942

[19] Bottari G, Ranieri VM, Ince C, et al: Use of extracorporeal blood purification therapies in sepsis: the current paradigm, available evidence, and future perspectives. Crit Care, 2024; 28: 432. 39722012 10.1186/s13054-024-05220-7 PMC11670469

